# Acute kidney injury-associated delirium: a review of clinical and pathophysiological mechanisms

**DOI:** 10.1186/s13054-022-04131-9

**Published:** 2022-08-27

**Authors:** Haoming Pang, Sanjeev Kumar, E. Wesley Ely, Michael M. Gezalian, Shouri Lahiri

**Affiliations:** 1 Departments of Neurology and Neurosurgery, Cedars-Sinai Medical Center, Los Angeles, CA 90048 USA; 2 Department of Nephrology, Cedars-Sinai Medical Center, Los Angeles, CA 90048 USA; 3 Division of Allergy, Pulmonary, and Critical Care Medicine, Vanderbilt University School of Medicine, Nashville, TN 37232 USA; 4 Departments of Neurology, Neurosurgery, and Biomedical Sciences, Cedars-Sinai Medical Center, Los Angeles, CA 90048 USA

## Abstract

Acute kidney injury is a known clinical risk factor for delirium, an acute cognitive dysfunction that is commonly encountered in the critically ill population. In this comprehensive review of clinical and basic research studies, we detail the epidemiology, clinical implications, pathogenesis, and management strategies of patients with acute kidney injury-associated delirium. Specifically addressed are the pathological roles of endogenous toxin or drug accumulation, acute kidney injury-mediated neuroinflammation, and acute kidney injury-associated volume overload as discrete potential biological mechanisms of the condition. The optimization of clinical contributors and normalization of renal function are reviewed as pragmatic management strategies in addition to potential and emerging therapeutic approaches.

## Introduction

Delirium occurs in approximately 60% of patients with acute kidney injury (AKI) [1]. Substantial clinical evidence suggests a direct pathological role for AKI in delirium. Studies demonstrate that the risk of delirium in patients with AKI significantly increases with worsening renal function [2, 3]. Although the underlying mechanisms for AKI-associated delirium remain largely unknown, several pathobiological processes have been proposed, including accumulation of neurotoxins or deliriogenic drugs due to impaired renal clearance, upregulation of systemic cytokine-mediated neuroinflammatory processes, and volume overloaded conditions.

The goal of this article is to provide a comprehensive review of the breadth of human and animal investigations that examine the clinical and pathophysiological contributions of AKI to delirium. This topic is of particular relevance as AKI affects up to half of critically ill patients [4] and may confer a tenfold increased risk of delirium [5], a condition known to increase morbidity and mortality, prolong hospital stay, and accelerate long-term cognitive decline [6, 7]. We further identify critical knowledge gaps in understanding of underlying biological mechanisms and clinical contributors to inform design of future studies that address novel preventative and therapeutic discoveries.

## Background and epidemiology

### AKI-associated delirium: clinical burden, long-term outcomes, and risks factors

AKI is estimated to affect more than 10 million people worldwide annually and confer a 1.7–6.9-fold increased risk of hospital mortality [8, 9]. Over half of all critically ill patients develop AKI within 48 h of admission to the intensive care unit (ICU) [4], with mounting preclinical evidence suggesting that AKI often precipitates or exacerbates secondary injury to other organ systems including the brain, heart, and lungs [10–13]. The most common clinical manifestation of AKI-associated acute brain injury is delirium, which presents as an acute or fluctuating impairment in attention, executive, function, or short-term memory [3, 14–18]. In the short term, delirium is well known to be strongly associated with increased mortality, prolonged hospitalization, and need for intensive medical interventions [1], while persistent cognitive decline is a feared long-term sequela [19].

The Bringing to Light the Risk Factors and Incidence of Neuropsychological Dysfunction in ICU Survivors (BRAIN-ICU) study found that patients with delirium who survived their hospital course developed long-term cognitive impairment including 20% of patients whose cognition was similar to that of Alzheimer’s disease [20]. Longer duration of delirium was also found to be an independent risk factor for worse global cognition [20, 21]. In terms of the public health impact, delirium accounts for significant societal and health care costs, with the national burden of delirium on the health care system costing up to $152 billion each year in the USA [22] as a result of prolonged hospital stay, increased treatment costs, and long-term acute care requirements [6]. It is now understood that delirium independently contributes to long-term cognitive decline [19], rather than merely an unmasking of a vulnerable brain substrate.

Several studies have identified AKI as a principal risk factor for delirium. In a prospective study of 1487 patients, Zipser et al. found that AKI conferred a tenfold increased risk of delirium (OR 10.01, CI 1.13–88.73, *p* = 0.039) [5], while the BRAIN-ICU study found that AKI was present in 50% of study days when patients were delirious [23]. Several studies have reported on a direct relationship between AKI severity and delirium risk. In one such study, AKI severity, as measured using the Kidney Disease: Improving Global Outcomes (KDIGO) creatinine criteria, was associated with a significantly increased risk of delirium. Specifically, a 1.5-fold increased risk of delirium was observed with KDIGO stage 2 (OR 1.55; 95% CI, 1.07–2.26) and a 2.5-fold increased risk of delirium with stage 3 (OR 2.56; 95% CI, 1.57–4.16) AKI, while KDIGO stage 1 was not significantly associated with delirium (OR 1.13, 95% CI 0.91–1.41) [2]. Concordant data were reported in a retrospective study that analyzed the medical records of 919 medical ICU patients of whom 41.6% developed AKI and found a higher incidence of delirium with KDIGO stage 2 (66.7%) and stage 3 (66.9%) AKI compared with KDIGO stage 1 (53.6%) [1]. Another prospective cohort study of 304 patients aged 60 or older found elevated creatinine level of > 2 mg/dL (OR 2.1, 95% CI 1.1–4.0) as one of 4 admission risk factors for delirium along with dementia, receipt of benzodiazepines before ICU admissions, and low arterial pH [24].

Further clinical evidence of AKI’s potential role in contributing to delirium was provided by Wan et al. who conducted a single-center case control study in a 30-bed mixed ICU in the UK with 142 cases and 142 matched controls to evaluate AKI-associated hyperactive delirium, a subtype of delirium. In this study, patients with KDIGO stage 3 AKI were five times more likely to develop hyperactive delirium (OR 5.40, 95% CI 2.33–12.51) than those without AKI and that less severe stages of AKI, i.e., KDIGO stage 1 or 2 AKI, were not independently associated with hyperactive delirium [25]. Overall, the dose-dependent relationship between severity of AKI and delirium identified by these studies suggests a direct pathological role, though causality cannot be reasonably established with clinical studies that may be susceptible to the presence of multiple confounding factors including, but not limited to, heterogeneities in use of analgosedation, identification of patients with increased susceptibility to delirium, such as those with preexisting cognitive impairment, and varied environmental triggers of delirium. Furthermore, additional challenges may be posed in differentiating between the more clinically apparent hyperactive from the often-missed hypoactive delirium phenotypes [26], which complicates clinical investigations on risk factors and mechanisms. Future studies should consistently report whether the investigated mechanisms or risk factors relate to hypoactive, hyperactive, or both delirium phenotypes.

A summary of clinical studies that have evaluated AKI as a risk factor in the development of delirium is presented in Table 1.

**Table 1:** Clinical studies investigating the association between acute kidney injury and delirium

Author(s)	Year	Methodology	AKI criteria	Strengths/Limitations	Conclusion
Wan et al.	2019	Case–control study 142 cases, 142 controls	KDIGO	Strengths: Matched case–control design using prevalent cases over a 1-year period. 97% specific for method to detect hyperactive delirium Limitations: Single-center study. Retrospective design limited to data recorded in electronic health records	AKI stage 3 is associated with hyperactive delirium (OR 5.40, 95% CI 2.33–12.51). AKI stages 1 and 2 were not independently associated with hyperactive delirium
Pisani et al.	2007	Prospective cohort (*n* = 304)	Serum creatinine level > 2 mg/dL	Strengths: At the time, the largest collection of data on delirium among older ICU patients. First to examine admission risk factors Limitations: Missing data on risk factors, specifically liver function test and arterial pH. Lack of generalization to younger population. Acute vs. chronic serum creatinine level > 2 was not distinguished as being associated with delirium	Serum creatinine level of > 2 mg/dL is an admission risk factor for delirium (OR 2.1, 95% CI 1.1–4.0)
Siew et al.	2017	Prospective cohort (*n* = 466)	KDIGO	Strength: Large sample size and prospective design. Findings persisted when using an alternative definition for AKI Limitations: Single-center population. Excluded patients with overt neurologic disorders. Did not have preadmission kidney function on all patients	AKI stage 2 (OR 1.55; 95% CI, 1.07–2.26) and stage 3 (OR 2.56; 95% CI, 1.57–4.16) are associated with delirium. AKI stage 1 was not significantly associated with delirium (OR 1.13, 95% CI 0.91–1.41)
Zipser et al.	2019	Prospective cohort (*n* = 1487)	Medical diagnoses data retrieved from the electronic medical chart (Klinikinformationssystem, KISIM, CisTec AG, Zurich) described as diagnostic clusters according to the 10^th^ revision of the International Classification of Diseases (ICD-10)	Strength: Systemically assessed predisposing and precipitating factors for delirium. Ample sample size Limitations: Selection of delirium-relevant ICD-10 codes chosen. Not all codes could be included and information may be skewed or lost in this process	AKI is associated with delirium (OR 10.01, CI 1.13–88.73, *p* = 0.039). Chronic kidney disease is not associated with delirium (OR 1.04, CI 0.61–1.78, *p* = 0.891)
Jäckel et al.	2021	Retrospective cohort (*n* = 919)	KDIGO	Strength: Adjusted for confounders of delirium thereby reducing bias Limitations: Used NuDesc to define delirium, hence definition not congruent to DSM-5 definition. Retrospective study. Baseline kidney functions not determined in all cases	Delirium is independently predicted by AKI stage 2/3 (OR 1.69, CI 1.04–2.73, *p* = 0.033)

AKI, Acute kidney injury; OR, odds ratio; CI, confidence interval; KDIGO, Kidney Disease: Improving Global Outcomes; and HR, hazards ratio

### Pathophysiology of AKI-associated delirium

**Fig. 1:**
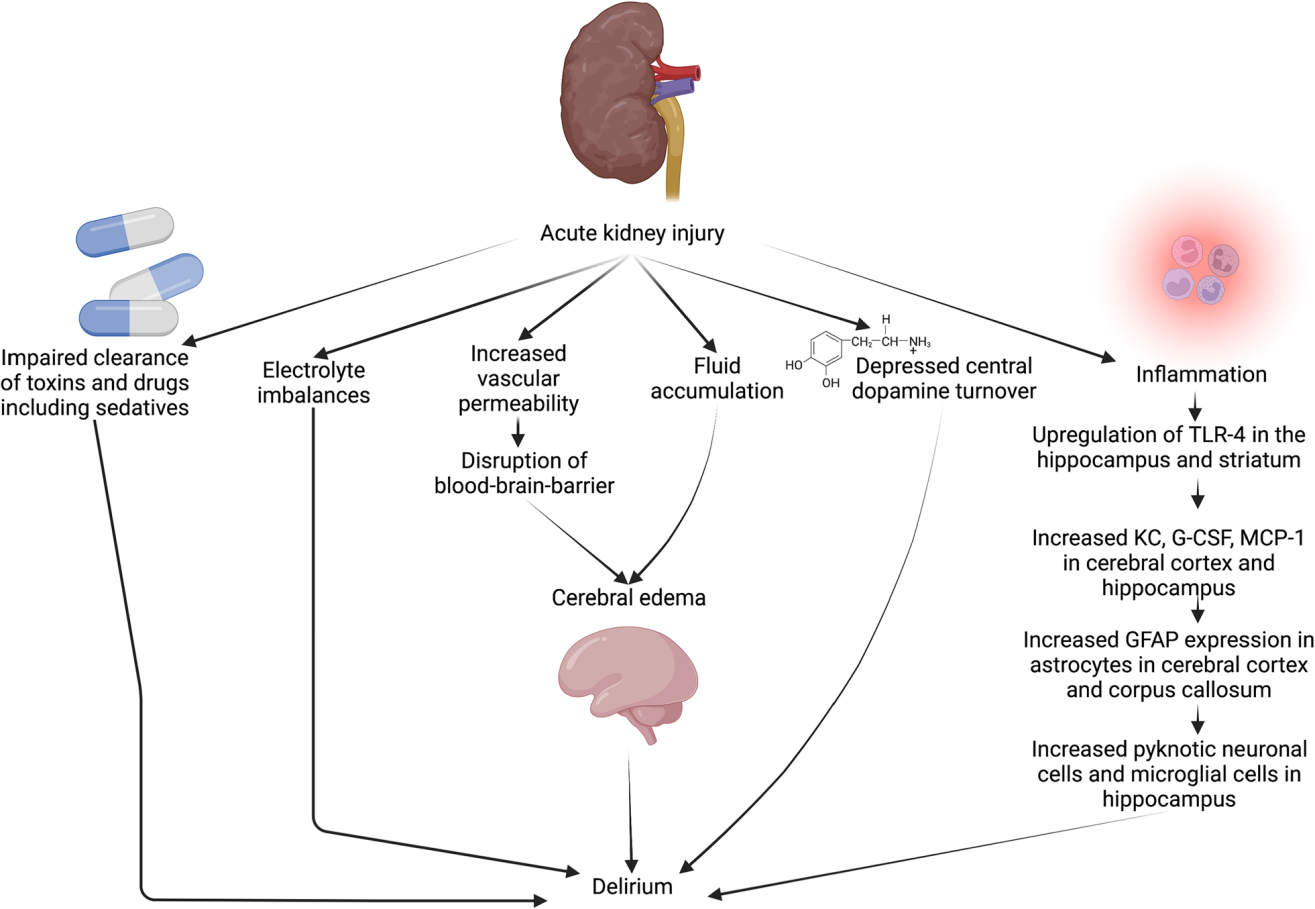
Proposed mechanisms of cognitive dysfunction as a result of acute kidney injury. TLR-4, toll-like receptor-4; KC, keratinocyte-derived chemokine; G-CSF, granulocyte colony-stimulating factor; MCP-1, monocyte chemoattractant protein-1; and GFAP, glial fibrillary acidic protein

Several studies have suggested AKI as a key contributor to distal organ dysfunction, not only affecting the brain, but also the heart, lungs, and liver [15, 27]. As with other organs affected by AKI, the pathogenesis of AKI-associated delirium is multifactorial, and hypothesized to be due to direct and indirect pathways, mediated by toxin and drug accumulations [25], electrolyte imbalances [28], impaired volume homeostasis [29], neuroinflammation [13], and imbalances in neurotransmitters [30] (Fig. 1).

### Direct neurotoxic effects of AKI from endogenous toxin accumulation

Perhaps intuitively, it is hypothesized that AKI precipitates delirium due to impaired renal clearance of drugs and toxic metabolic waste products. A potential explanation for the direct neurotoxic effect of AKI is from the accumulation of potential uremic neurotoxins. While urea may be considered a surrogate for accumulated neurotoxins, it is not thought to directly contribute to delirium [31]. Although over 130 substances are considered to be potential uremic toxins [32], the uremic guanidino compounds, which include creatinine, guanidine, guanidinosuccinic acid, and methylguanidine, are considered particularly relevant to the pathogenesis of delirium [15, 33, 34]. These compounds are hypothesized to exert their neurotoxic effects through the inhibition of ϒ-aminobutyric acid receptors and activation of *N*-methyl-d-aspartate (NMDA) receptors which results in neuronal hyperexcitability, abnormal epileptiform activity, and hippocampal injury [33]. While mouse models demonstrate that administration of exogenous creatinine precipitated epileptic activity, these effects were far greater with other guanidino compounds, specifically guanidinosuccinic acid [35].

Given the risk of uremic compounds in inducing neuronal hyperexcitability [33, 35], seizure should be considered in the evaluation of delirium in the setting of AKI. However, it is often clinically challenging, if not impossible, to distinguish between delirium and non-convulsive seizures, due to shared clinical phenotypes and precipitants. Additionally, kidney injury, both acute and chronic, may lead to electrolyte and metabolic disorders like hyponatremia, hypocalcemia, hypomagnesemia, or hypoglycemia that can independently precipitate epileptic activity [36]. Clinically, one case–control study by Oddo et al. found that at least chronic kidney disease was significantly associated with periodic epileptiform discharges. Although AKI did not show statistical significance, there was a trend toward increased risk of periodic epileptiform discharges with AKI (26% with AKI vs 19% without, *p* = 0.21) [37]. These findings suggest a potential role for electroencephalographic monitoring in AKI-associated delirium to evaluate for seizures as a treatable etiology of delirium-like states, particularly in patients with overt seizure-like semiology, myoclonus, or forced eye gaze deviation.

### Direct neurotoxic effects of AKI from drug accumulation

Another common explanation for delirium in the setting of AKI is the accumulation of drugs that are frequently administered in the ICU setting. A common mechanistic theme for the deliriogenic drugs, such as benzodiazepines and certain antibiotics, is their ϒ-aminobutyric acid antagonistic properties [38–42].

Cefepime-induced neurotoxicity is a relatively commonly precipitant of delirium, imposing up to a tenfold greater risk of neurotoxicity when compared to meropenem, and occurring in up to 15% of ICU patients treated with cefepime [42]. The setting of critical illness is believed to create an inflammatory environment that disrupts the integrity of the blood–brain barrier (BBB) [43], thus allowing for the penetration of cefepime into the brain. Given that cefepime is renally cleared, AKI further exacerbates cefepime-induced neurotoxicity due to drug accumulation [42]. If delirium due to cefepime-induced neurotoxicity is suspected, one should investigate for the presence of non-convulsive status epilepticus, which occurs in a quarter of such patients [42]. Adjustment of cefepime dosing or avoidance of cefepime is recommended in AKI to prevent neurotoxicity manifesting as delirium.

Certain classes of drugs, such as opioids and neuropathic agents, confer various degrees of delirium, largely based on their anticholinergic properties. Meperidine, for instance, should be avoided in AKI because its metabolite, normeperidine, may accumulate and result in central nervous system excitation, induce life-threatening seizures, and exacerbate the phenotype of delirium [44–46]. An expanded discussion on the risks of delirium from analgosedatives in the setting of AKI will be reviewed below.

### AKI-associated systemic and brain inflammation

There is mounting preclinical evidence that AKI induces systemic inflammation, which is considered a key contributing mechanism of delirium [47]. Data from animal models suggest that AKI promotes upregulation of systemic inflammatory processes that contribute to endothelial injury, leukocyte infiltration, release of cytokines and inflammatory mediators, and induction of apoptosis [25, 48, 49]. This pro-inflammatory milieu precipitated by AKI is hypothesized to contribute to multi-organ injury, including the brain [50–52]. There is an increased systemic production of interleukin-1 α (IL-1α), IL-1β, IL-6, IL-10, and tumor necrosis factor α (TNF-α), which are implicated in the pathogenesis of delirium [13]. Other animal studies suggest that AKI-induced systemic and neuroinflammation contributes to BBB disruption and altered expression of tight-junctional proteins, resulting in the infiltration of metabolites and toxins into the brain and leading to inflammatory and pathological changes in the brain [34, 53]. This is evidenced by mouse models of AKI that demonstrated extravasation of Evans blue dye into the brain, indicating breakdown of the BBB [13]. In the clinical setting, this process of increased brain vascular permeability, microvascular protein leakage, and alterations of aquaporins allows for metabolites and toxins that are normally impermeable to the BBB to injure the brain and result in cerebral edema commonly seen in AKI patients [48, 49, 54, 55]. Clinical evidence of BBB disruption serving a pathophysiologic role in delirium is provided in one study that showed elevated serum levels of S100β, the marker of BBB damage [56] in elderly patients with delirium [57].

The pro-inflammatory cytokines, IL-6, TNF-α, IL-1α, IL-1β, have been implicated in delirium-like behavioral changes, such as impaired concentration, diminished motivation, and psychomotor retardation in critically ill patients [52, 58–60]. Among various cytokines, IL-6 has been frequently studied as a potential predictor of delirium in urinary tract infection, sepsis, acute lung injury, and perioperative animal models [53, 61–66]. Indeed, animal studies have revealed that IL-6 is both necessary and sufficient to produce cognitive decline [67]. It is postulated that surgical intervention may induce neuroinflammation and contribute to cognitive decline. For instance, it has been found that orthopedic surgery disrupts the BBB and promotes infiltration of bone marrow-derived monocytes [68] and activation of microglia [69] in rodents. This is concordant with clinical studies that have found that high levels of IL-6 preoperatively were significantly associated with postoperative delirium in patients admitted for elective and emergency surgery [62, 70]. Given that these pro-inflammatory cytokines, especially IL-6, have also been demonstrated to be elevated in AKI, similar mechanisms for delirium in AKI are likely, but remain to be proven.

Animal models demonstrate that the structural areas of the brain disproportionately affected by AKI-induced inflammatory mediators include the CA1 region of the hippocampus [13], which is consistent with the semiology of delirium and the hippocampus’ established involvement in learning and memory as well as anxiety and depression [71]. Furthermore, the CA1 neurons of the hippocampus are vulnerable to damage in several other conditions that result in neurodegeneration, including global cerebral ischemia, Alzheimer’s disease, and prion diseases [72–74].

**Fig. 2:**
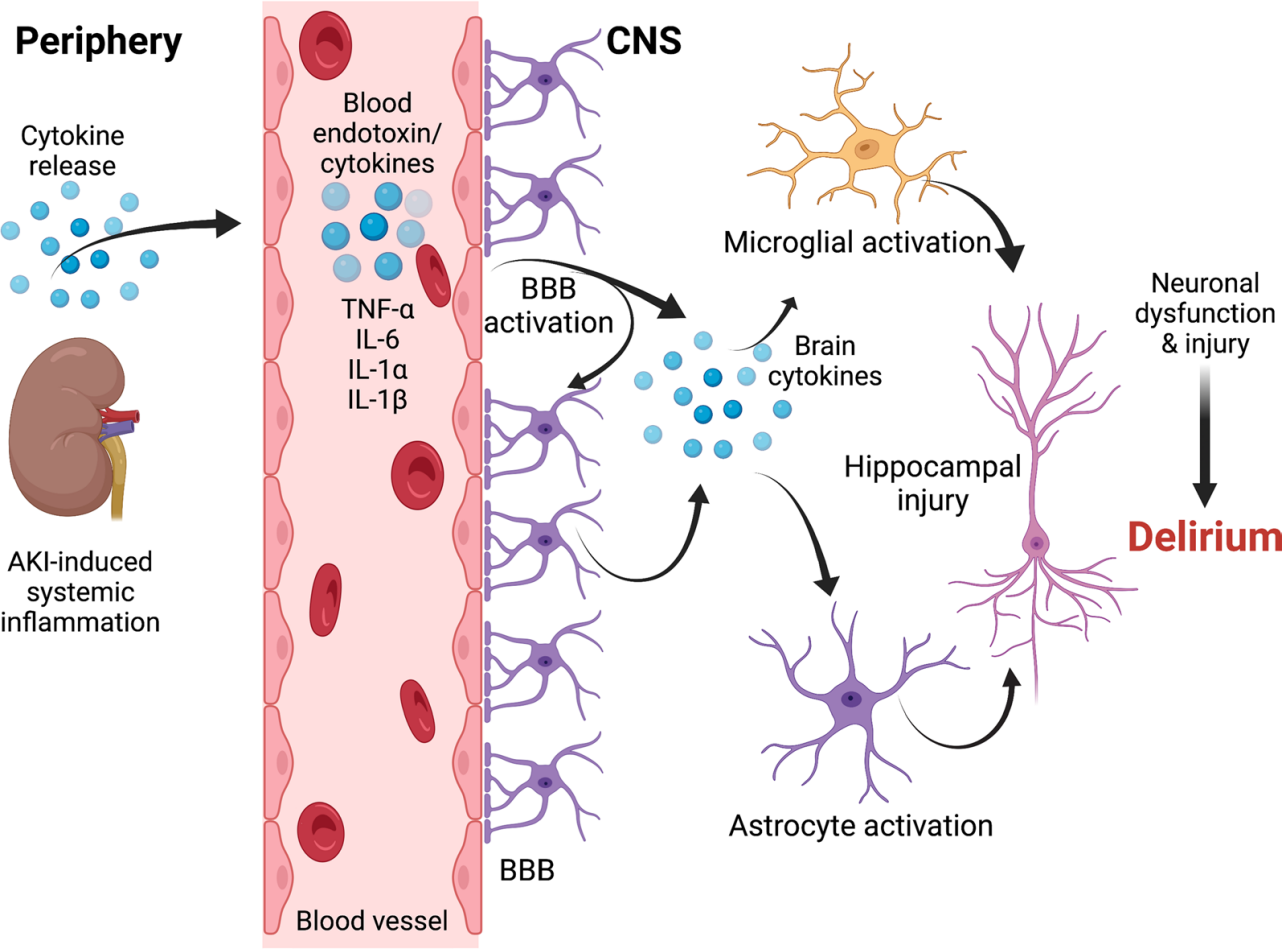
Post-AKI microglial and astrocyte activation as potential cellular drivers of delirium. AKI, acute kidney injury; TNF, tumor necrosis factor; IL, interleukin; and BBB, blood–brain barrier

Mouse models of AKI [13], prion disorders [75], and Alzheimer’s disease [76] all suggest that hippocampal CA1 pathology is in part, accountable for hypoactivity in mice. Hippocampal injury and inflammation highlighted by pyknotic neuronal cells [77], activation of microglial cells [13], upregulation of toll-like receptor-4 [78], increased levels of keratinocyte-derived chemokine, and increased levels of granulocyte colony-stimulating factor [79] within the hippocampus of renal ischemia reperfusion injury-induced AKI mouse models provide further evidence for a direct pathological role for AKI in delirium. Other areas of the brain involved include the cerebral cortex and the corpus callosum as evidenced by astrogliosis [13], a marker for activated glial cells during brain inflammation [80, 81]. Thus, the activation of central immune cells leading to neuronal injury and dysfunction [82] may contribute to post-AKI delirium (Fig. 2).

Cerebral injury from AKI may potentially be reversible if mild in severity or if treated early, as is the case with the clinical course of delirium. This point was suggested by findings by Liu et al. who found no neuronal apoptotic changes in mice with AKI as evidenced by minimal terminal deoxynucleotidyl transferase-mediated digoxigenin-deoxyuridine nick-end labeling staining and minimal caspase-3 signaling on immunostaining [13].

A summary of experimental studies on brain effects of inflammation (Table 2) is shown below.

**Table 2:** Summary of experimental studies on the effects of inflammation on the central nervous system

Author(s)	Year	Model	Conclusion
Rashid et al.	2021	UTI in mice Readout: behavioral and structural brain dysfunction	Mice with UTI demonstrated impairments of the frontal cortex and hippocampus, which were reversed following treatment with systemic anti-IL-6 antibody
An-HsunChou et al.	2014	60 min bilateral ischemia reperfusion injury-induced AKI Study endpoint/s: 2 and 24 h Readout: cDNA based microarray	Mice with AKI exhibited upregulated mRNA levels of genes involved in inflammation
Salama et al.	2013	Bilateral renal ischemia reperfusion injury in rats Readout: TLR-4 expression	↑ TLR-4 expression within hippocampus and striatum
Liu et al.	2008	60 min bilateral ischemia reperfusion injury-induced AKI Study endpoint: 24 h Readout: Histology	↑ Neuronal pyknosis and microgliosis ↑ Keratinocyte-derived chemoattractant and G-CSF in the cerebral cortex and hippocampus ↑ Expression of glial fibrillary acidic protein in astrocytes in the cortex and corpus callosum
Adachi et al.	2001	Bilateral rat renal artery occlusion. Endpoint: 48 h Readout: motor activity and brain monoamine turnover	↓ Turnover of DA in the striatum, mesencephalon and hypothalamus Impaired motor activity

UTI, urinary tract infection; cDNA, complementary deoxyribonucleic acid; TLR-4, toll-like receptor-4; AKI, acute kidney injury; IL-6, interleukin-6; mRNA, messenger RNA; G-CSF, granulocyte colony-stimulating factor; and DA, dopamine

### AKI-associated fluid overload

Another potential mechanism of AKI-associated delirium is fluid overload, which can occur in 40% of patients in the ICU [83]. Fluid overload is thought to increase capillary transmural hydrostatic pressure, resulting in fluid leak into brain interstitial tissue causing cerebral edema [83]. Using a multivariate proportion odds logistic regression model for delirium in mechanically ventilated patients, a retrospective observational cohort study found that fluid overloaded patients, defined as when the recorded body weight was 10% higher than at baseline, resulted in more delirium days (OR 2.16, 95% CI 1.05–4.47) [83].

Concordantly, a study by Nguyen et al. found that fluid overload was independently associated with development of delirium in patients with shock (171 ± 104 in the delirium group vs. 128 ± 80 ml/kg; both *p* = 0.001) [29]. Interestingly, this study did not find a difference in central venous pressure between the two groups, suggesting that delirium due to fluid overload is independent of venous congestion; however, central venous pressure is associated with increased risk of AKI [84]. Nguyen et al. hypothesized that mechanism behind delirium from fluid overload was from brain vasogenic edema due to BBB leakage as evidenced by increased serum S100β [29], which is an early marker of BBB disruption [85]. BBB leakage thus promotes brain edema and allows for movement of neurotoxic substances into the brain [86], resulting in delirium. Thus, a reasonable approach to fluid status in a patient with AKI is to avoid hypervolemia, which not only protects the kidneys from further injury [87], but also protects the brain. Further investigations into the cognitive effects of fluid overload in the setting of AKI are warranted.

### Hormonal and neurotransmitter effects of AKI

AKI may also lead to changes in the hormonal balance and neurotransmitter turnover in the brain which may contribute to delirium. Adachi et al. studied changes in the monoamine metabolism and motor activity in AKI rats [30] and found an overall decrease in dopamine turnover in the striatum, mesencephalon, and hypothalamus of AKI rats, while the turnover of norepinephrine or 5-hydroxyindoleacetic acid, the main metabolite of serotonin, was not affected by AKI. The authors postulated that the impairment of spontaneous motor activity in the AKI rats, a sign of delirium, may be related to depressed central dopamine turnover [30], resulting in impairments with memory, learning, anxiety, and depression [88–90].

Additionally, neurologic abnormalities from AKI may be related to the rise of calcium content in the brain [28]. This hypothesis comes from studies of AKI in canines, which identified biochemical alternations in the brain, whereby calcium contents in gray and white matter markedly increased 3 days after the onset of AKI along with a modest increment of magnesium in delirium-relevant regions of the brain. While these increases were thought to be related to excess parathyroid hormone [28], it is known that hypercalcemia is one of the reversible metabolic causes of delirium in patients with advanced cancer for instance, and that treatment of hypercalcemia resulted in symptom control [91]. Thus, for patients with delirium in the setting of AKI, it is reasonable to evaluate and treat hypercalcemia as a contributing factor.

### Potential therapeutic treatments of AKI-associated delirium

There is a pressing need for novel clinical interventions to ameliorate AKI-associated delirium. With the possible exception of renal replacement therapy, existing treatment paradigms are limited to lower, indirect evidence of benefit. A summary of the potential therapeutic approaches to AKI-associated delirium is provided in Table 3.

**Table 3:** Summary of evidence-based therapies, potentially useful therapies, and emerging therapies to prevent AKI-associated delirium

Direct evidence-based therapies [23]	Indirect evidence-based therapies [44, 100, 110, 113, 117]	Emerging therapies and future investigations [66, 96, 97, 116]
Study: Prospective cohort Siew et al. 2017 Findings: Renal replacement therapy is associated with a reduced odds of delirium in AKI Recommendations: Future studies are needed to examine the mechanisms underlying these associations and the effects renal replacement therapy may have on AKI-associated delirium. Consider the use of renal replacement therapy to reduce risk of delirium	Study: Systematic review and meta-analysis on dexmedetomidine Flükiger et al. 2018 Findings: Overall incidence of delirium in the dexmedetomidine group was significantly lower compared to placebo, standard sedatives, and opioids Recommendations: Preferential use of dexmedetomidine for sedation, which may also potentially be renally protective (Liu et al.)	Study: Study on the use of anti-IL-6 to reverse delirium-like phenotypes in mice model of UTI Rashid et al. 2021 Findings: Mice with UTI had significantly elevated plasma IL-6 and demonstrated behavioral impairments that were fully reversed with treatment with systemic anti-IL-6 Recommendations: Given that anti-IL-6 reversed UTI-induced delirium-like phenotypes in mice, future studies should examine if systemic or targeted IL-6 inhibition may also mitigate AKI-associated delirium
	Study: Systematic review of the risk of delirium with different opioids Swart et al. 2017 Findings: Significant risk of delirium from the use of meperidine, compared with other opioids. Decreased risk with hydromorphone and fentanyl Recommendations: Preferential use of fentanyl compared to other opioids in setting of renal impairment	Study: Study of the effects of GSA intrahippocampal injection in rats Pan et al. 1996 Finding: GSA-injected animals led to seizures and damage to the hippocampus that was prevented by the administration of the NMDA receptor antagonist ketamine Recommendations: Given that GSA is increased in the serum and CSF of patients with renal failure, future studies may examine the use of NMDA receptor antagonists to prevent delirium in AKI
	Study: Review paper on use of Drugs in End-Stage Kidney Disease Wilcock et al. 2017 Findings: Lower risk of accumulation with lorazepam compared to diazepam or clonazepam in ESRD Recommendations: When benzodiazepines are indicated, lorazepam may be preferred	Study: Retrospective cohort Murugan et al. 2021 Findings: Lower rates of ultrafiltration reduced organ dysfunction Recommendations: Future studies are needed to determine the effects of lower rates of ultrafiltration on delirium in patients with AKI
	Study: Case–control study Lieberman et al. 1985 Findings: For patients with chronic renal failure, use of tricyclic antidepressants leads to elevated serum levels of glucuronidated metabolites Recommendations: Given that glucuronidated metabolites of tricyclic antidepressants were reported to exert potent biologic effects peripherally and in the central nervous system (Lieberman et al.), a similar accumulation of metabolites may occur in AKI, potentially leading to delirium, although future research is needed to prove this. Minimizing the use of neuropathic agents, which may accumulate in the context of AKI, may decrease delirium risk	

AKI, Acute kidney injury; ESRD, end-stage renal disease; CKD, chronic kidney disease; CNS, central nervous system; GSA, guanidinosuccinic acid; CSF, cerebrospinal fluid; and NMDA, *N*-methyl-d-aspartate

#### Renal replacement therapy

A prospective observational cohort study found that renal replacement therapy modifies the risk of AKI-associated delirium. Specifically, among patients not on renal replacement therapy, an increase in daily peak serum creatinine of 1 mg/dl was significantly associated with increased odds of delirium (OR, 1.35; 95% CI, 1.18–1.55), whereas patients receiving renal replacement therapy, daily peak serum creatinine was not associated with delirium (OR, 1.07; 95% CI, 0.87–1.31). The authors hypothesized that renal replacement therapy diminishes the effects of AKI on the brain by clearing neurotoxic sedatives, antibiotics, and metabolites [2]. Although early initiation of renal replacement therapy may shorten length of stay in the intensive care [92] or in-hospital settings [93], it is unclear if improved delirium outcomes drive this effect. Prior randomized clinical trials were not designed to assess delirium as a primary outcome, leaving opportunities for future clinical trials to evaluate the potential for both benefits and risks of invasive renal replacement therapy [92, 94].

Ultrafiltration may be an intuitive approach to addressing AKI-associated delirium, due to fluid overload; however, any potential benefits of ultrafiltration need to be balanced with the concerns related to cerebral hypoperfusion [95] and worsened renal recovery [96]. Although delirium has not been specifically evaluated in prior studies using ultrafiltration, prior studies have shown improved rates of extracerebral organ dysfunction with lower compared to higher rates of ultrafiltration [96]. Future studies are needed to examine the viability of ultrafiltration in hypervolemic patients with AKI to mitigate delirium.

Additionally, data from the United States Renal Data System found the risk of incident dementia to be lower in patients on peritoneal dialysis compared to hemodialysis, with a hazard ratio 0.74 (95% CI 0.64–0.86) in a matched model. Cognitive impairment rates for patients undergoing hemodialysis are 1.5–2.0 times higher than for those undergoing peritoneal dialysis [97]. This was suggested to be attributable to a reduction in the hemodynamic instability and rapid changes in cerebral blood flow typically seen with patients undergoing hemodialysis [90]. Future studies are needed to assess whether renal replacement therapy strategies that lower the risk of hemodynamic instability potentially mitigate the risk of delirium.

#### Analgosedation optimization

Sedatives, such as benzodiazepines and opiates, are known to be highly deliriogenic and may potentially exacerbate delirium in the context of AKI. Careful consideration of their respective metabolism and clearance pathways is warranted when choosing between various sedatives in the setting of AKI.

Metabolites of commonly used benzodiazepines, for example, the active midazolam metabolite, α-hydroxymidazolam, are cleared by the kidneys and accumulate with renal failure [98], which may prolong the duration of their pharmacological effects [23]. In contrast, the lorazepam metabolite, lorazepam glucuronide, is a nontoxic metabolite and its drug clearance is not altered by renal disease [99]. As such, studies from patients with end-stage renal disease report lower risk of accumulation with lorazepam compared to midazolam, diazepam, clonazepam [100, 101]. Despite the relative renal safety of lorazepam, a side effect of the solvent used in intravenous lorazepam can produce propylene glycol toxicity, which can lead to proximal tubular necrosis resulting in AKI, in addition to metabolic acidosis, serum hyperosmolality, and elevated anion gap [102]. Patients with AKI may be at higher risk of propylene glycol accumulation, although it is reassuring that while in one study, propylene glycol toxicity occurred in 19% of medical ICU patients receiving high-dose lorazepam infusion, none had any significant clinical deterioration [103].

Similar considerations related to the mechanism of drug elimination are justified when using opioids in patients with AKI. Opioids that undergo significant clearance through the kidneys should be avoided. For instance, the metabolites of tramadol, morphine, codeine, and meperidine are renally cleared [45, 104] and thus justify caution or dose adjustment in the setting of AKI. In particular, the morphine metabolite, morphine-6-glucuronide, has prolonged effects in the brain once it crosses the BBB, and even with discontinuation or dialyzing to remove the metabolite, the brain effects persist for some time as morphine-6-glucuronide re-equilibrates across the BBB [105]. Additionally, a systematic review by Swart et al. on the comparative risk of delirium with different opioids found the highest risk of delirium with meperidine and tramadol, likely due to the high anticholinergic properties of the opioids and their metabolites [44].

In contrast, fentanyl is extensively metabolized via CYP3A4 into norfentanyl, which is an inactive metabolite, and methadone is ultimately metabolized into pyrroline, which can be eliminated through feces [104]. The risk of delirium was noted to be lower with fentanyl compared to other opioids [44], findings similar to that of a systematic review by King et al. which noted less harm in renally impaired patients when using fentanyl compared to other opioids, such as morphine [106]. In fact, fentanyl use has been associated with reduced delirium [83]; thus, in patients with AKI, given the safety profile with renal dysfunction as well as relatively low risk of delirium, fentanyl should be the first-line opioid when indicated.

Neuropathic agents, such as tricyclic antidepressants, are known contributors to delirium—perhaps mediated by their anticholinergic effects [107]. Thus, such agents must be used with caution in patients with renal disease given that serum levels of glucuronidated metabolites of tricyclic antidepressants accumulate with kidney dysfunction. Other neuropathic agents such as gabapentin and pregabalin must also be used cautiously in the setting of AKI since these agents are renally excreted. The toxic accumulation of such drugs may result in depressed mental status [108] and mimic delirium.

Dexmedetomidine, an α-2 adrenergic agonist, may be superior to other sedatives such as benzodiazepines, propofol, and opioids [109–111] given its lack of ϒ-aminobutyric acid properties, as with benzodiazepines and propofol, or anticholinergic properties, such as with opioids, both of which are thought to play a role in the pathogenesis of delirium. Dexmedetomidine may also provide a more natural sleep-like sedation pattern, which may reduce the risk of developing delirium [110]. Dexmedetomidine’s delirium-sparing effects in AKI may also be related to its hepatic clearance [112], and potential renoprotective effects [113] via stabilization of the sympathetic system, and anti-inflammatory toll-like receptor-4-mediated effects [113]. Therefore, in the setting of AKI, it seems reasonable to use dexmedetomidine over other types of sedatives [114].

#### Potential emerging therapies and future investigations

In the future, immunomodulating therapies may play a role in the prevention and treatment of AKI-associated delirium. Although various cytokines and inflammatory mediators are upregulated in AKI, recent animal studies suggest that the use of systemic IL-6 inhibition mitigates delirium-like phenotypes in urinary tract infection [66], acute lung injury [65], and postoperative states [67]. Given that IL-6 is significantly upregulated in AKI [115], future studies are needed to determine whether modulation of the IL-6 signaling pathway mitigates AKI-associated delirium.

As noted earlier, uremic guanidino compounds may play a central role in AKI-associated delirium by promoting NMDA receptor-mediated depolarization of hippocampal neurons, therefore minimizing epileptiform discharges, and hippocampal damage [33]. In a study using rodents, ketamine, an NMDA receptor antagonist, prevented epileptiform activity and hippocampal damage induced by injection of guanidinosuccinic acid, one of the key neuroanatomical structures believed to be affected in AKI-associated delirium [116]. Therefore, future studies are needed to assess the role of NMDA receptor antagonists to prevent or treat AKI-associated delirium. While studies using NMDA receptor antagonists to reduce postoperative delirium have yielded mixed results, further research is needed to assess the role of NMDA receptor agonists specifically in AKI-associated delirium.

## Conclusion

The pathogenesis of AKI-associated delirium is multifactorial and includes both inflammatory- and non-inflammatory-mediated processes, such as the accumulation of toxins and drugs, structural brain injury from systemic inflammation, impaired volume homeostasis, and hormonal and neurotransmitter effects. Current evidence suggests that, when possible, gradual normalization of kidney function may ameliorate delirium; however, optimization of other clinical contributors, such as deliriogenic drugs, including analgosedation and antibiotics, provides additional opportunities to mitigate delirium. Future investigations are needed to understand the role of systemic immunomodulation to ameliorate AKI-associated delirium.

## Data Availability

Data in this study were a review of existing data, which are openly available at locations cited in the reference section.
